# The Emergency Management and Treatment of Severe Burns

**DOI:** 10.1155/2011/161375

**Published:** 2011-09-04

**Authors:** Melanie Stander, Lee Alan Wallis

**Affiliations:** Division of Emergency Medicine, Stellenbosch University, Cape Town 7505, South Africa

## Abstract

Burn injuries continue to cause morbidity and mortality internationally. Despite international collaborations and preventative measures, there are still many cases reported in high- and low-income countries. The treatment of these patients is often protracted and requires extensive resources. The adequate resuscitation of these patients coupled with meticulous wound care can have a huge impact on their outcome. The authors present a simple guideline for the initial management of severe burns which is utilised by the South African Burn Society and is based on the guidelines of the American Burn Association and the Australian and New Zealand Burn Association.

## 1. Introduction

Burn wounds and injuries are often devastating. They can have severe long-term consequences for the victims and they continue to be a major problem affecting communities worldwide [1]. The treatment of these patients is often protracted, and large amounts of resources are often needed to achieve the medical and psychological healing that needs to occur. Prevention is the vital factor that will have an impact on decreasing the morbidity and mortality associated with burns [2–4]. Education and training are vital steps to empower communities to help them protect themselves, and also the most vulnerable of burn victims are children. There have been studies into the different epidemiological factors related to burn injuries [5–11] with the subsequent introduction of training programmes, community outreach and social development, and the development of safe and effective household practices. These include initiatives like the Global Alliance for Clean Cookstoves [12]. International organisations like the World Health Organisation's Department of Violence and Injury Prevention and Disability (VIP) and the International Society for Burns Injuries (ISBI) strive to ultimately decrease this significant scourge by improving data collection, research collaborations, and preventative strategy development [13]. 

Statistics from the WHO demonstrate that there are over 300,000 deaths per year from fires alone with many more from scalds, electrical burns, and other sources but there is still no accurate global data to confirm these numbers [13]. Over 95% of fatal fire-related burns occur in low- and middle-income countries [13]. Multitudes more patients have survived their injuries but are often left disfigured and destitute. Children and the elderly remain the most vulnerable groups with the highest mortality [13]. Intensive and specialised burn centres are in existence all over the world but are very often situated in high-income countries. These innovative and expensive treatment modalities play an important part, but the way in which a burn patient is initially managed carries an equally important role. Simple adherence to the basics including adequate resuscitation and meticulous wound care go a long way to achieving favourable outcomes and even in influencing mortality rates [14]. The following guidelines are based on the South African Burn Society management guidelines [15] which in turn are based on the American Burn Association [16] and Australian and New Zealand Burn Association guidelines [17].

## 2. Minimal Criteria for Transfer to a Burn Centre

Burn injury patients who should be referred to a burn unit include the following:

- all burn patients less than 1 year of age;
- all burn patients from 1 to 2 years of age with burns >5% total body surface area (TBSA);
- patients in any age group with third-degree burns of any size;
- patients older than 2 years with partial-thickness burns greater than 10% TBSA;
- patients with burns of special areas—face, hands, feet, genitalia, perineum or major joints;
- patients with electrical burns, including lightning burns;
- chemical burn patients;
- patients with inhalation injury resulting from fire or scald burns;
- patients with circumferential burns of the limbs or chest;
- burn injury patients with preexisting medical disorders that could complicate management, prolong recovery, or affect mortality;
- any patient with burns and concomitant trauma;
- paediatric burn cases where child abuse is suspected;
- burn patients with treatment requirements exceeding the capabilities of the referring centre;
- septic burn wound cases.

## 3. Treatment Protocol

### 3.1. Remove any Sources of Heat

1. Remove any clothing that may be burned, covered with chemicals, or that is constricting.
2. Cool any burns less than 3 hours old with cold tap water (18 degrees centigrade is adequate) for at least 30 minutes and then dry the patient.
3. Cover the patient with a clean dry sheet or blanket to prevent hypothermia.
4. Use of Burnshield [18] is a very effective means of cooling and dressing the injury for the first 24 hours.
5. Rings and constricting garments must be removed.

### 3.2. Assess Airway/Breathing

1. Careful airway assessment must be done where there are flame or scald burns of the face and neck. Intubation is generally only necessary in the case of unconscious patients, hypoxic patients with severe smoke inhalation, or patients with flame or flash burns involving the face and neck. Indications for airway assessment include the presence of pharyngeal burns, air hunger, stridor, carbonaceous sputum, and hoarseness.
2. All patients with major burns must receive high-flow oxygen for 24 hours.
3. Always consider carbon monoxide poisoning in burn patients. They may have the following symptoms: restlessness, headache, nausea, poor co-ordination, memory impairment, disorientation, or coma. Administer 100% oxygen via a non-rebreathing face mask; if possible, measure blood gases including carboxyhaemoglobin level.
4. If breathing seems to be compromised because of tight circumferential trunk burns, consult with the burn centre surgeons immediately regarding the need for escharotomy.

**Circulation**

1. Stop any external bleeding.
2. Identify potential sources of internal bleeding.
3. Establish large-bore intravenous (IV) lines and provide resuscitation bolus fluid as required in all compromised patients, using standard ATLS protocols [19]. Perfusion of potentially viable burn wounds is critical.

**Figure 1:**
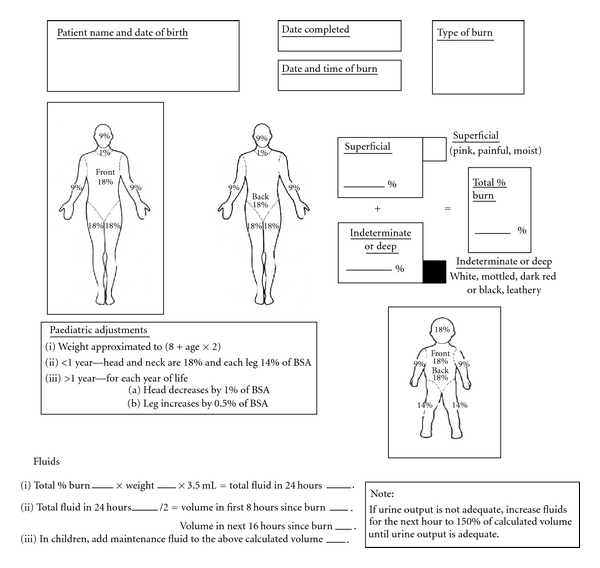
South African Burn Society Burn Assessment Form.

**Estimate the Percentage Total Body Surface Area (%TBSA) Burned (See Figure 1)**

Initially, use the Rule of Nines. In the case of all paediatric patients and for a more accurate assessment, use the Berkow diagram; alternatively, the patient's unstretched open hand represents 1% of TBSA.

**Reminder**

Accurate estimation of burn size is critical to ongoing fluid replacement and management.

### 3.3. Ongoing Losses (Once the Patient Has Been Stabilised)

1. Patients with <10% TBSA burns can be resuscitated orally (unless the patient has an electrical injury or associated trauma). This needs ongoing evaluation and the patient may still require an IV line.
2. In the case of patients with burns 10–40% TBSA, secure a large-bore IV line; add a second line if transportation will take longer than 45 minutes.
3. Burns >40% TBSA require 2 large-bore IV lines.
4. If the transfer will take less than 30 minutes from the time of call, do not delay transfer for an IV line.

**Reminder**

IV lines may be placed through the burned area if necessary (suture to secure). Avoid the saphenous vein if at all possible, and avoid cut-downs through unburned skin if possible. An intraosseous line is an excellent alternative in children.

(5) Initiate fluids for ongoing resuscitation and fluid losses using the Parkland formula
  (1) $$\begin{array}{cc} & 4\;\text{mL}\;\text{crystalloid}\times \left(\text{kg of body weight}\right)\\ & \;\times \left(\%\text{burn}\right)=\text{mL}\;\text{in}\;\text{first}\;24\;\text{hours}, \end{array}$$
  with half of this total given in the first 8 hours after injury (note that this is the time from burn, not from presentation to healthcare services). Children must have their daily maintenance fluids added to these replacement fluids (including dextrose).

**Example 3.1**

In the case of a patient weighing 70 kg with a 50% TBSA burn, (4 × 70 × 50) = 14 000 mL needed in the first 24 hours. Half is needed in the first 8 hours after injury.

**Example 3.2**

The fluid requirements of a child weighing 15 kg with a TBSA burn of 40% (4 × 15 × 40) = 2400 mL in the first 24 hours plus maintenance requirements of 1250 mL (1000 mL + 250 mL) = 3650 mL in the first 24 hours. Half is needed in the first 8 hours after injury.

**Reminder**

Do not give dextrose solutions (except for maintenance fluids in children)—they may cause an osmotic diuresis and confuse adequacy of resuscitation assessment. Ideally, use Ringer's lactate or normal saline for replacement fluid and a 5% dextrose-balanced salt solution for the child's maintenance.

*This is only a guide, and ongoing evaluation is essential as patients may need more fluids than calculated. Use the patient's vital signs and, most importantly, urine output to guide ongoing requirements.*

### 3.4. Assess Urine Output (This Is the Best Guide to Resuscitation)

1. Insert a Foley catheter in patients with burns >15% TBSA. Adequate urine output is 0.5 mL/kg/h in adults and 1.5 mL/kg/h in children.

**Reminder**

Lasix and other diuretics must not be given to improve urine output; increase IV fluid rates to increase urine output.

(2) Observe urine for burgundy colour (seen with massive injuries or electrical burns). There is a high incidence of renal failure associated with these injuries, requiring prompt and aggressive intervention.

**Reminder**

If the urine is red or brown consult a burn centre.

### 3.5. Insert a Nasogastric Tube

Insert a nasogastric tube in any patient with burns >30% TBSA, or any patient who is unresponsive, shocked, or with burns >20% if preparing for air or long-distance transportation.

### 3.6. Decompression Incisions (Escharotomy)

Assess for circumferential full-thickness burns of the extremities or trunk. Elevate the burned extremities on pillows above the level of the heart. If transfer will be delayed, discuss indications and methods for decompression incisions (escharotomies) with a burn surgeon.

### 3.7. Medication

1. Give tetanus immunisation.
2. After fluid resuscitation has been started, pain medication may be titrated in small intravenous doses (not intramuscular). Blood pressure, pulse, respiratory rate, and state of consciousness should be assessed after each increment of IV morphine.

### 3.8. Wound Care

1. Debridement and application of topical antimicrobials are usually unnecessary. Initial wound care needs to ensure that the burn is kept covered and the patient is kept warm. Plastic food wrap (such as Gladwrap) is ideal.
2. Apply a thin layer of silver sulfadiazine to open areas if transportation will be delayed for more than 12 hours.
3. Use of Burnshield is a very effective means of cooling and dressing the injury in the first 24 hours.

### 3.9. General Items

1. A history, including details of the accident and preexisting diseases/allergies, should be recorded and sent with the patient.
2. Copies of all medical records, including all fluids (calculation of fluids administered) and medications given, urine outputs, and vital signs must accompany the patient. These specific details may be recorded on the back of the burn size assessment sheet.
3. The burn centre will arrange transport if appropriate.
4. In the case of paediatric patients not accompanied by a parent, obtain consent in consultation with your burn centre.

### 3.10. Special Considerations with Chemical Burns (Consult Burn Centre)

(1) Remove all clothing.
(2) Brush powdered chemicals off the wound, then flush chemical burns for a minimum of 30 minutes using copious volumes of running water. Be careful to protect yourself.

**Reminder**

Never neutralise an acid with a base or vice versa; the heat generated can worsen the burn.

(3) Irrigate burned eyes using a gentle stream of saline. Follow with an ophthalmology consultation if transportation is not imminent.
(4) Determine what chemical (and what concentration) caused the injury.

### 3.11. Special Considerations with Electrical Injuries (Consult Burn Centre)

1. Differentiate between low-voltage (<1000 v) and high-voltage (>1000 v) injuries.
2. Attach a cardiac monitor; treat life-threatening dysrhythmias as needed.
3. Assess for associated trauma; assess central and peripheral neurological function.
4. Administer Ringer's lactate; titrate fluids to maintain adequate urine output or to flush pigments through the urinary tract (see urine output above). Useful laboratory test: arterial blood gas levels with acid/base balance.
5. Using pillows, elevate burned extremities above the level of the heart. Monitor distal pulses.
